# Supplementary material for: The unconventional activation of the muscarinic acetylcholine receptor M4R by diverse ligands
Source: Nat Commun. 2022 May 23;13:2855. doi: 10.1038/s41467-022-30595-y (PMC9126879; doi:10.1038/s41467-022-30595-y)
Supplement: Supplementary file 1 — Supplementary information [file 41467_2022_30595_MOESM1_ESM.pdf]

Supplementary information

Supplemental Figures

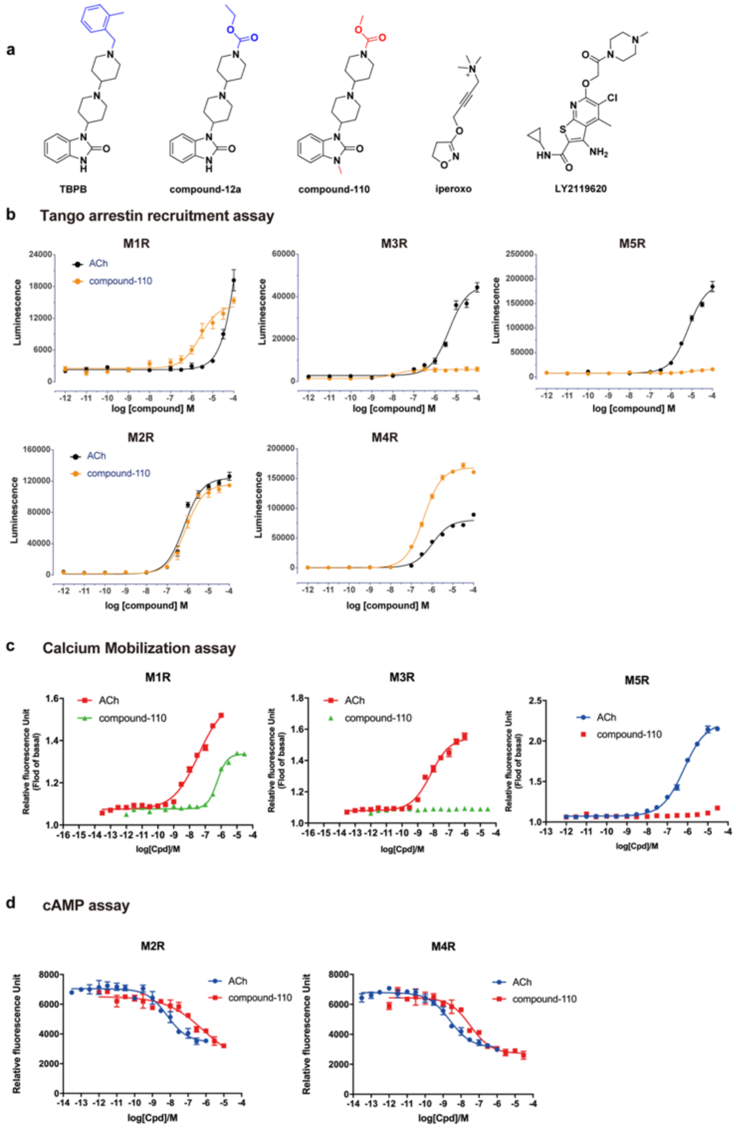

Supplementary Figure 1. The characterization of compound-110's selectivity within muscarinic receptors (M1R-M5R). a) The chemical structures of TBPB, compound-12a, compound-110, iperoxo and LY2119620. **b)** The tango assays for compound-110 within muscarinic receptors (M1R-M5R), data are represented as mean ± SEM from two independent assays with two technical replicates. **c)** The calcium mobilization assay in G_q signal pathway for compound-110 among M1R, M3R, and M5R, data are represented as mean ± SEM from one independent assay with three technical replicates. **d)** The cAMP assay in G_i signal pathway for compound-110 between M2R and M4R, data are represented as mean ± SEM from one independent assay with three technical replicates. Source data are provided as a Source Data file.

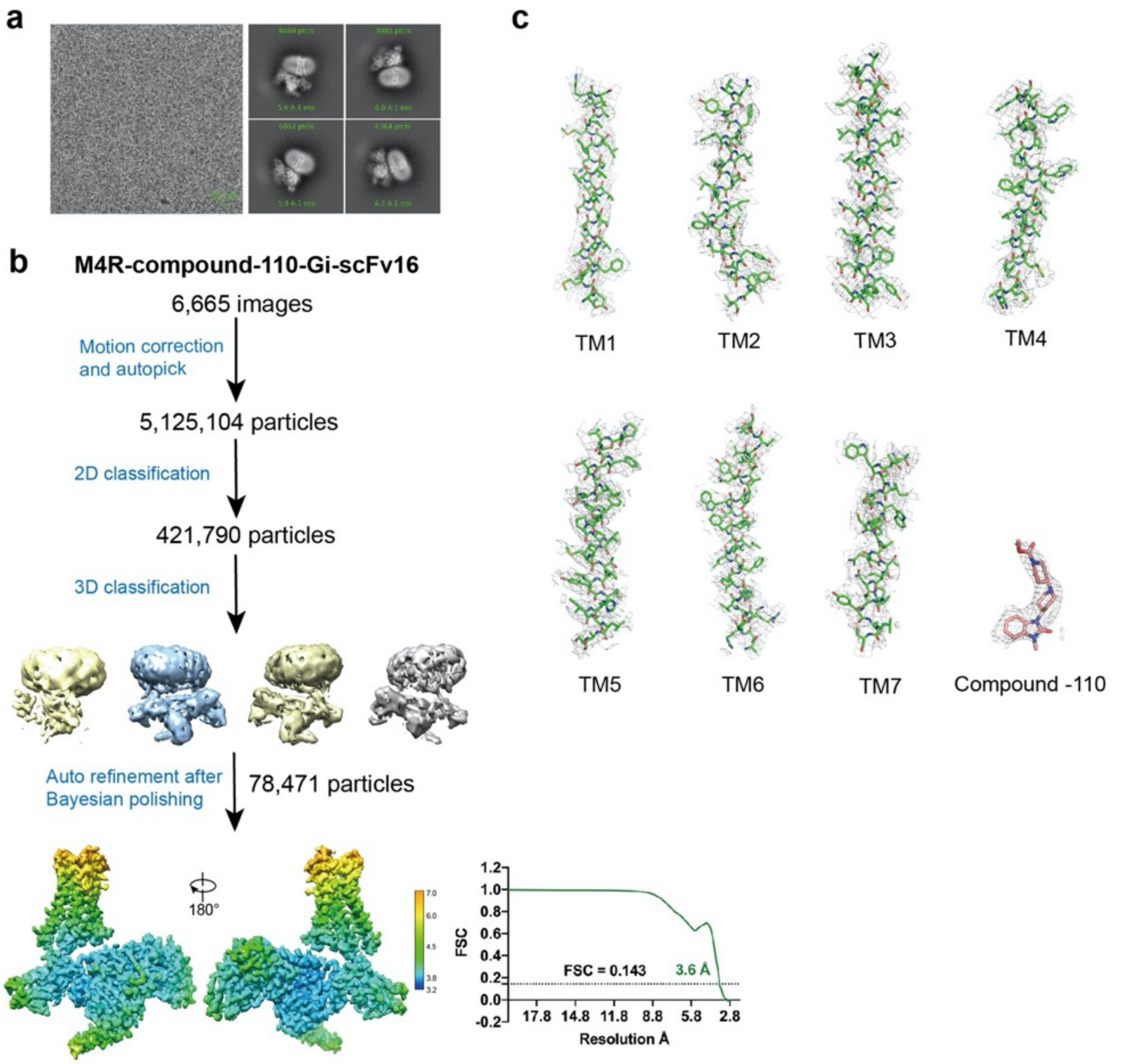

Supplementary Figure 2. Flowchart of cryo-EM data analysis of the M4R-c110-G$_i$ complex. **a)** Representative cryo-EM image (from 6665 images) of the M4R-c110-G$_i$-scFv16 complex (scale bar: 30 nm) and representative 2D classification showing distinct structural features of each component. **b)** M4R-c110-G$_i$ data processing and refinement (left) with 'Gold-standard' Fourier shell correlation curves (bottom inset) indicating resolution at FSC = 0.143 is 3.6 Å. **c)** Cryo-EM density maps and models of the M4R-c110-G$_i$ complex are shown for all transmembrane helices and compound-110.

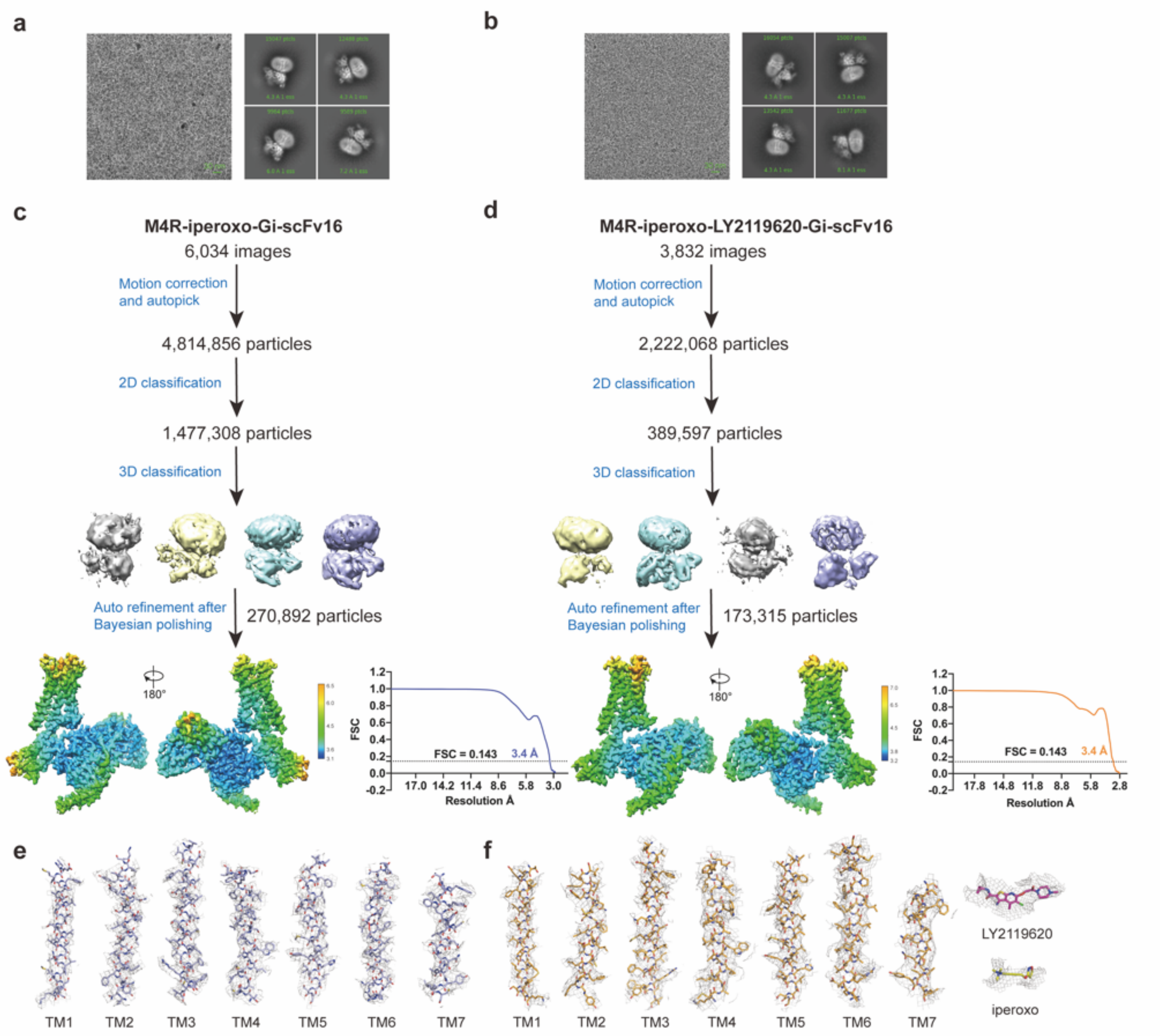

Supplementary Figure 3. Flowchart of cryo-EM data analysis of M4R-ip-G_i and M4R-ip-LY-G_i complexes. a and b) Representative cryo-EM images (scale bar: 30 nm) (from 6034 and 3832 images, respectively) and representative 2D classification showing distinct structural features of each component for the M4R-ip-G_i-scFv16 (A) and M4R-ip-LY-G_i-scFv16 (B) complexes. **c)** M4R-ip-G_i data processing and refinement (left) with 'Gold-standard' Fourier shell correlation curves (bottom inset) indicating resolution at FSC = 0.143 is 3.4 Å. **d)** As in A for M4R-ip-LY-G_i with resolution at FSC = 0.143 is 3.4 Å. **e and f)** Cryo-EM density maps and models of the M4R-ip-G_i (e) and M4R-ip-LY-G_i (f) complex, are shown for all transmembrane helices and ligands.

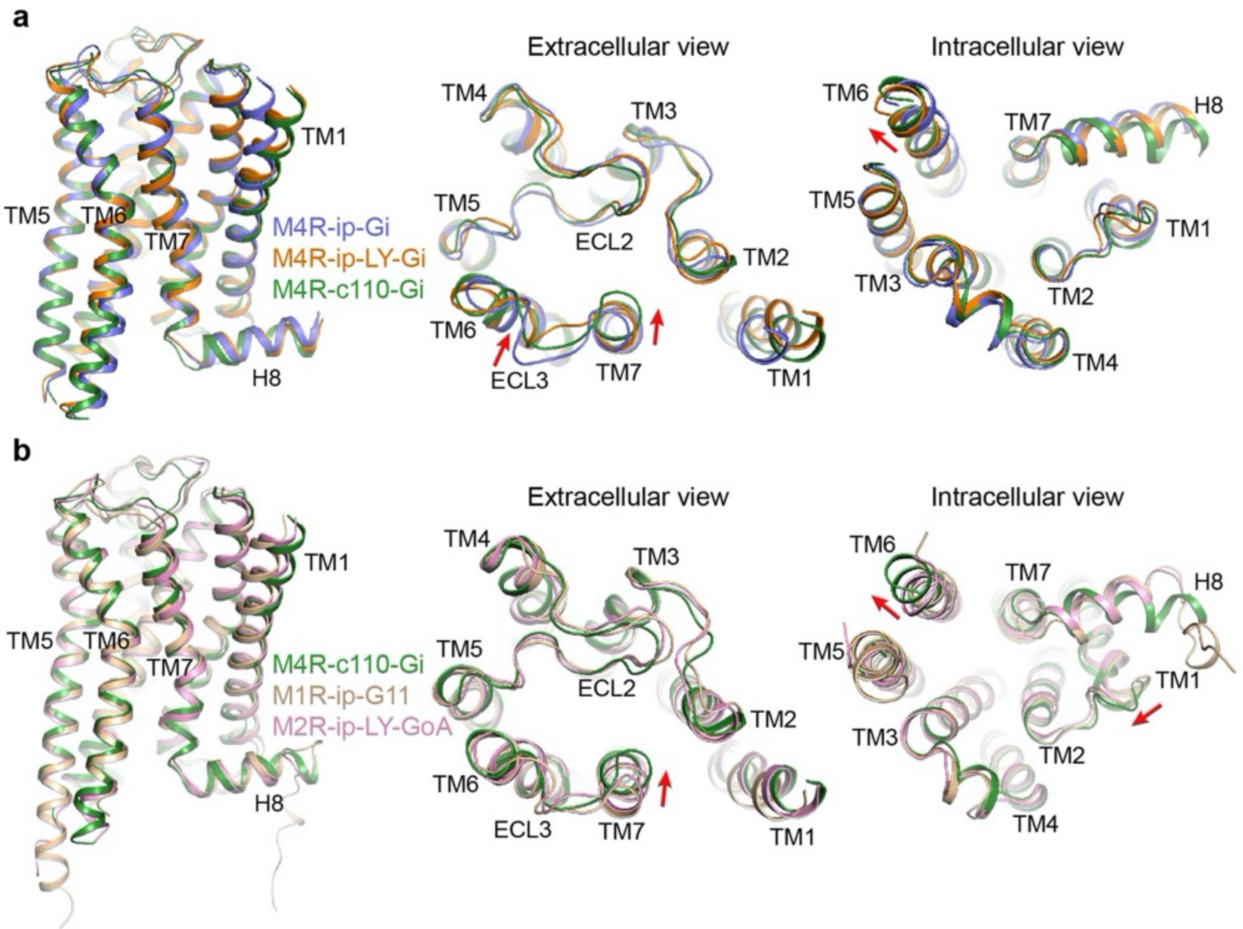

Supplementary Figure 4. Structural comparison of active M4R, M1R and M2R structures. a) Overall comparison of the active states of M4R in the extracellular and intracellular views. **b)** The comparisons active M4R-c110-Gi with M1R and M2R active structures, and shown in the extracellular and intracellular views, separately.

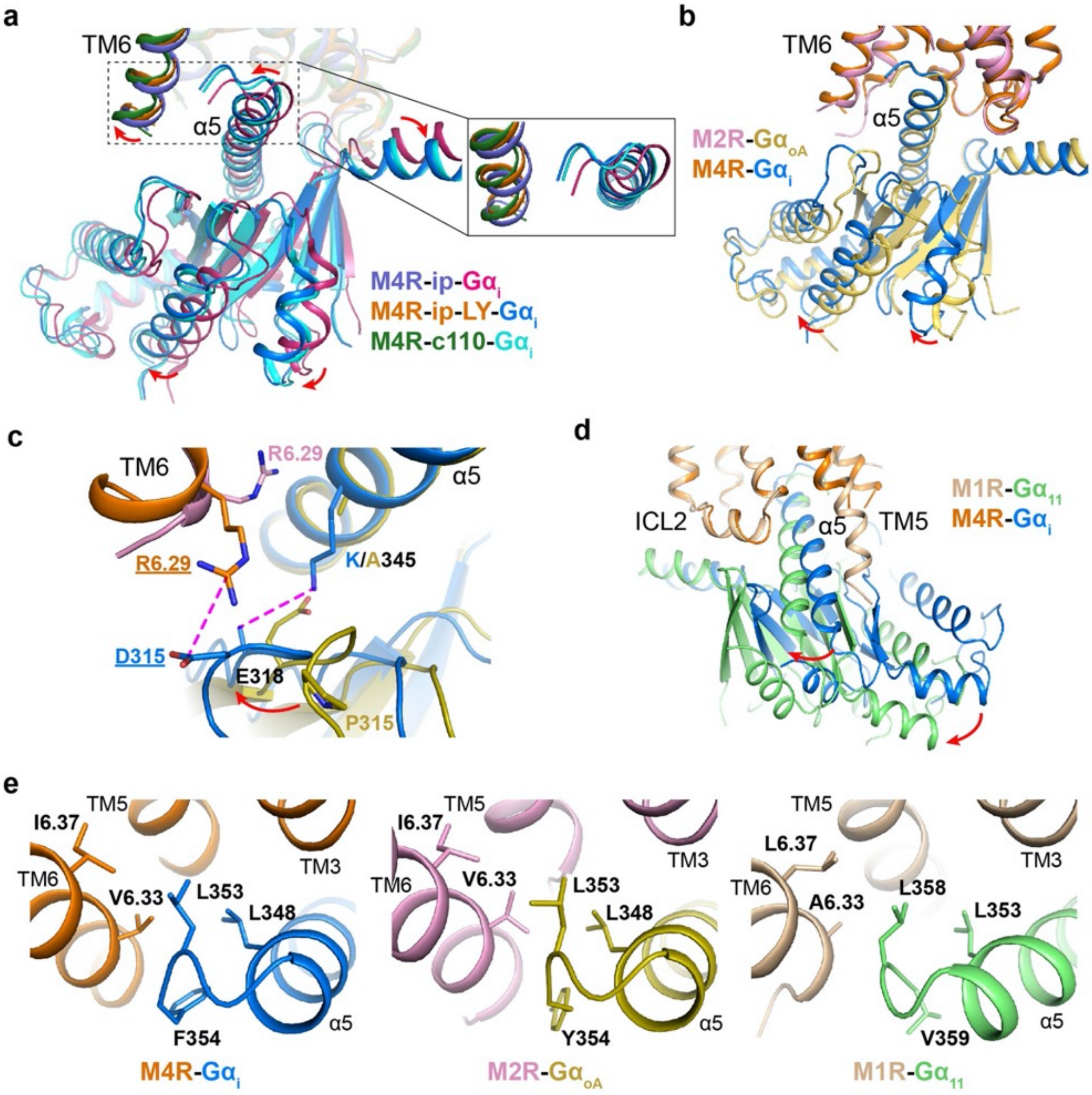

Supplementary Figure 5. Comparison of the receptor-G protein binding interfaces in solved mAChR-G protein complexes. a) Comparison of the receptor-G protein interface in M4R-ip-G_i, M4R-ip-LY-G_i, and M4R-c110-G_i complex structures when the receptors are aligned. **b**) Comparison of the M4R-ip-LY-G_i and M2R-G_{oA} complex interfaces when the receptors are aligned. **c)** Interaction differences of the TM6-Gα interface between M4R-ip-LY-G_i and M2R-G_{oA} structures. **d**) Comparison of the M4R-ip-LY-G_i and M1R-G_{11} complex interfaces when the receptors are aligned. **e**) Comparison of α5/TM6 interactions in M4R-ip-LY-G_i, M2R-G_{oA}, and M1R-G_{11} complex structures.

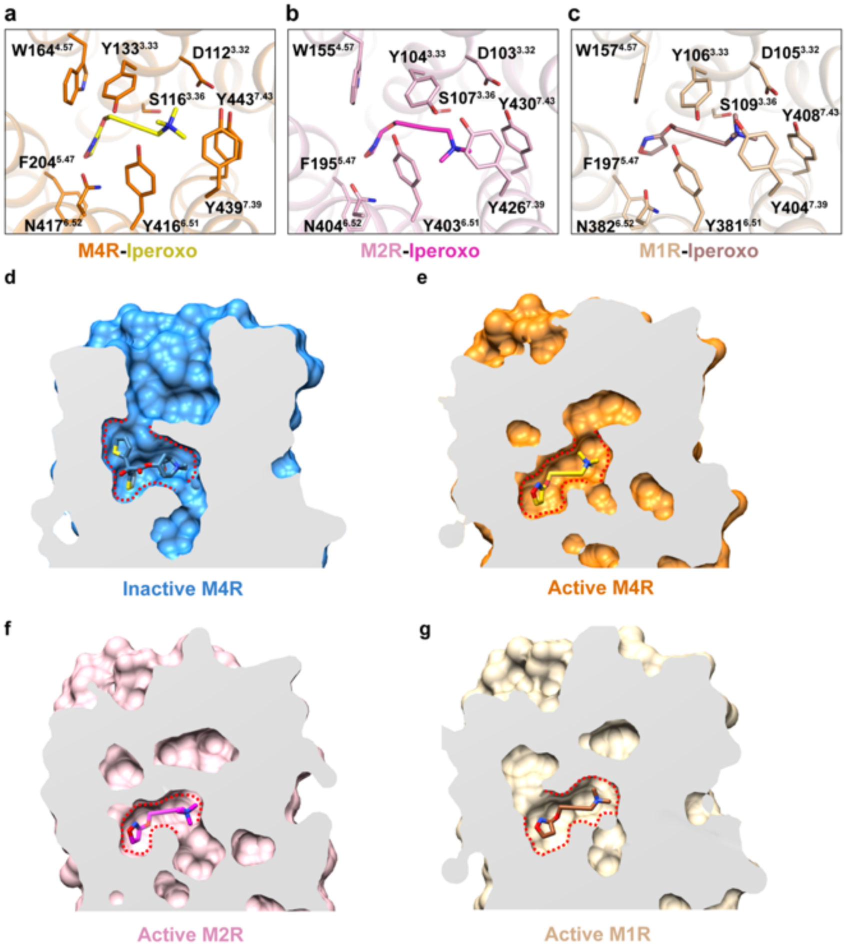

Supplementary Figure 6. Comparison of iperoxo binding pockets in M4R, M2R, and M1R active structures. a, b and c) Sliced view of the iperoxo binding pockets in various receptor structures. **d, e, f, and g)** Key residues involved in iperoxo binding are shown as sticks.

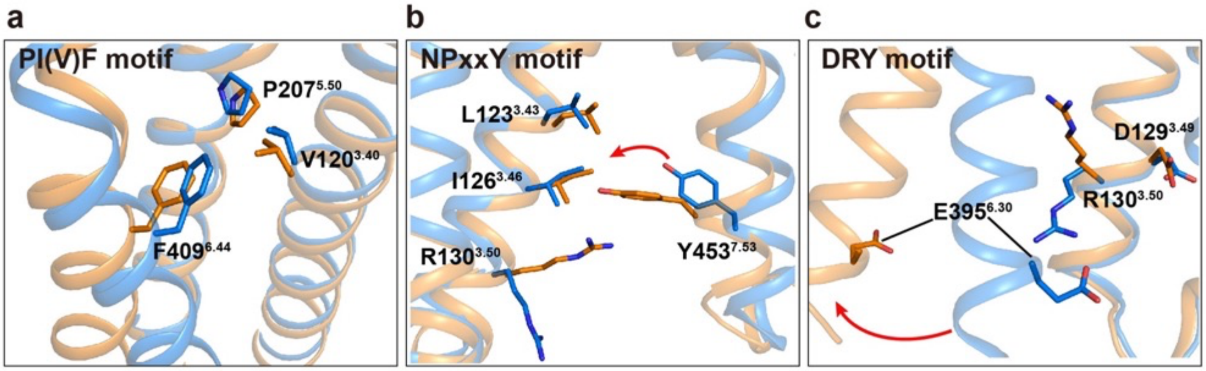

Supplementary Figure 7. Comparison of activation motifs between inactive M4R (PDB code, 5DSG, blue) and active M4R-ip-LY-G_i (orange) structures. a) PI(V) F motif, **b)** NPxxY motif and **c)** DRY motif. The red arrows indicate the conformational change from inactive- to active- states.

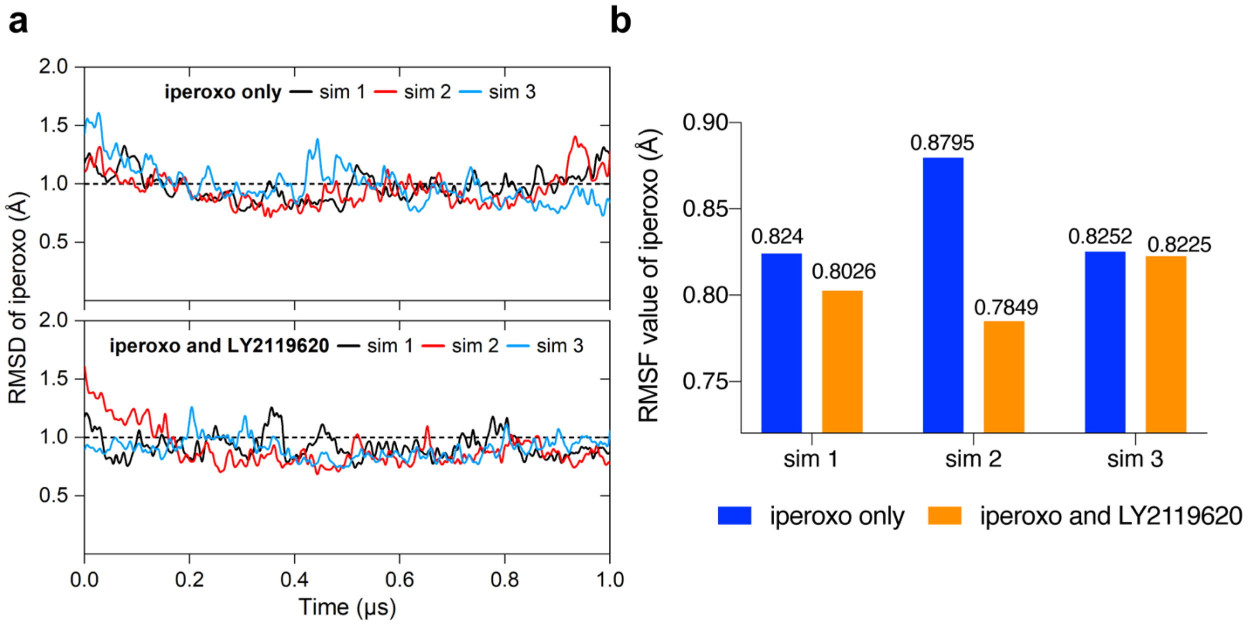

Supplementary Figure 8. The cooperativity of iperoxo and LY2119620 in M4R in MD simulations. Three independent MD simulations runs (1 us each) performed on M4R from M4-ip-Gi and M4-ip-LY-Gi complexes, separately. RMSD and RMSF values are calculated for each run. a) RMSD values of Iperoxo molecule in M4-ip (top) and M4-ip-LY (bottom) in three runs, sim1, sim2 and sim3. b) RMSF values of Iperoxo molecule in M4-ip (blue) and M4-ip-LY (orange) in three runs, sim1, sim2 and sim3. Source data are provided as a Source Data file.

Supplementary Figure 9. The G protein and β-arrestin recruitments assays of iperoxol, ACh, compound-110 and LY2119620 in M4R and the cooperativity of compond-110 and LY2119620 with Ach in the BRET assays. Values are shown as the mean ± SEM from n = 4 independent biological replicates, each biological replicate has two technical replicates. Effects of the mutations of key residues on compound-110 induced cAMP accumulation. data are represented as mean ± SEM from one independent assay with three technical replicates. Source data are provided as a Source Data file.

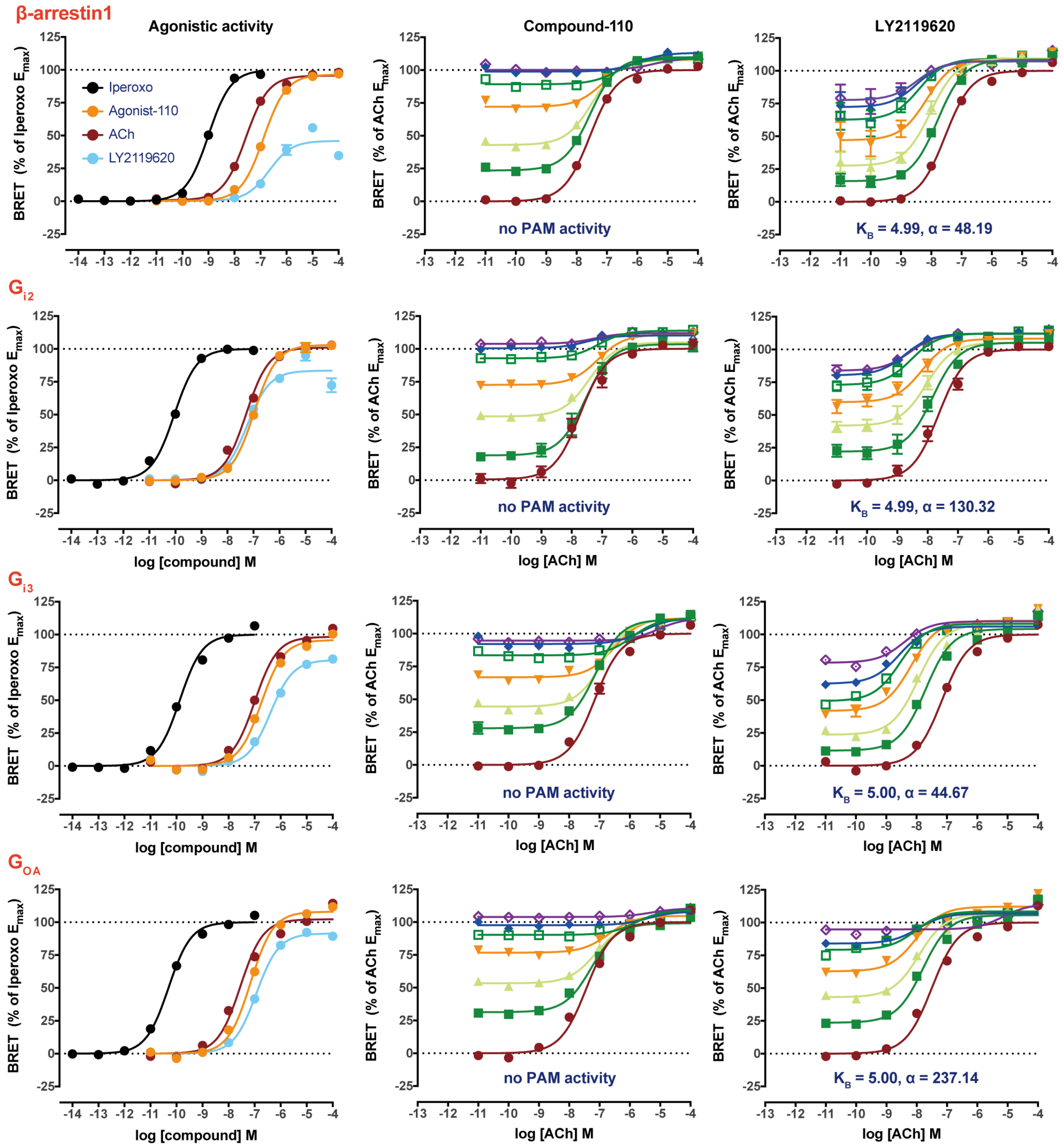

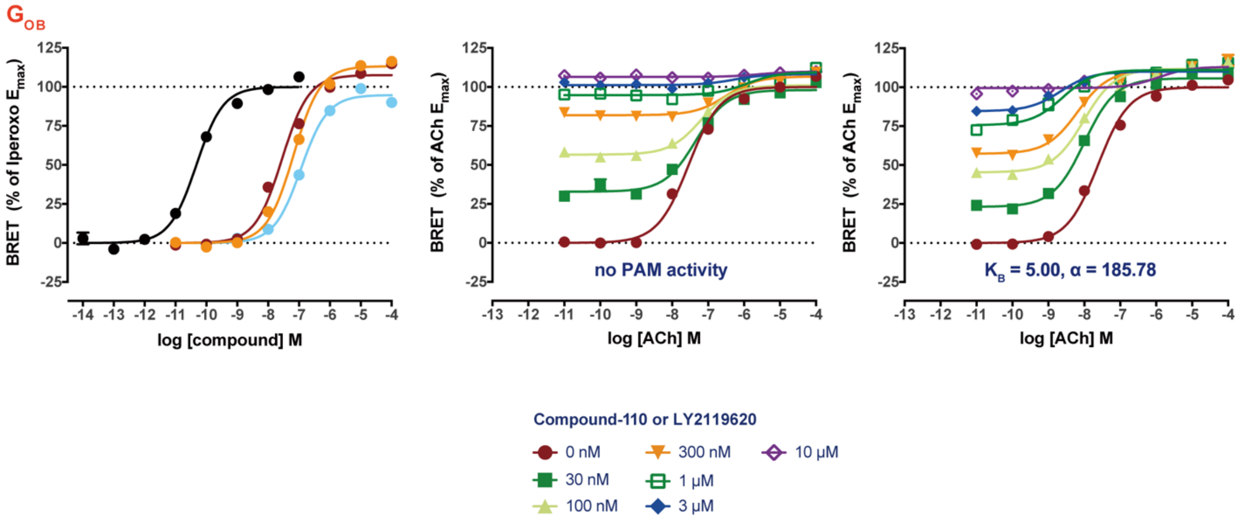
GOB
BRET (% of Iperoxo Emax)
log [compound] M
BRET (% of ACh Emax)
no PAM activity
log [ACh] M
BRET (% of ACh Emax)
KB = 5.00, α = 185.78
log [ACh] M
Compound-110 or LY2119620
0 nM
300 nM
10 μM
30 nM
1 μM
100 nM
3 μM

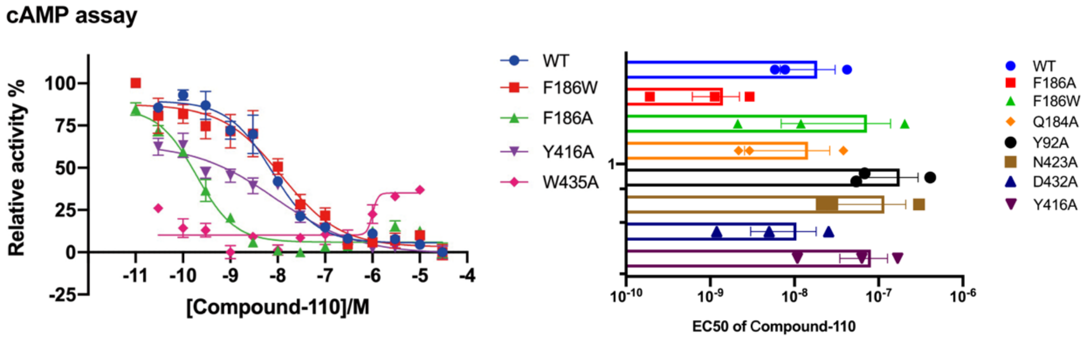
cAMP assay
Relative activity %
[Compound-110]/M
WT
F186W
F186A
Y416A
W435A
EC50 of Compound-110
WT
F186A
F186W
Q184A
Y92A
N423A
D432A
Y416A

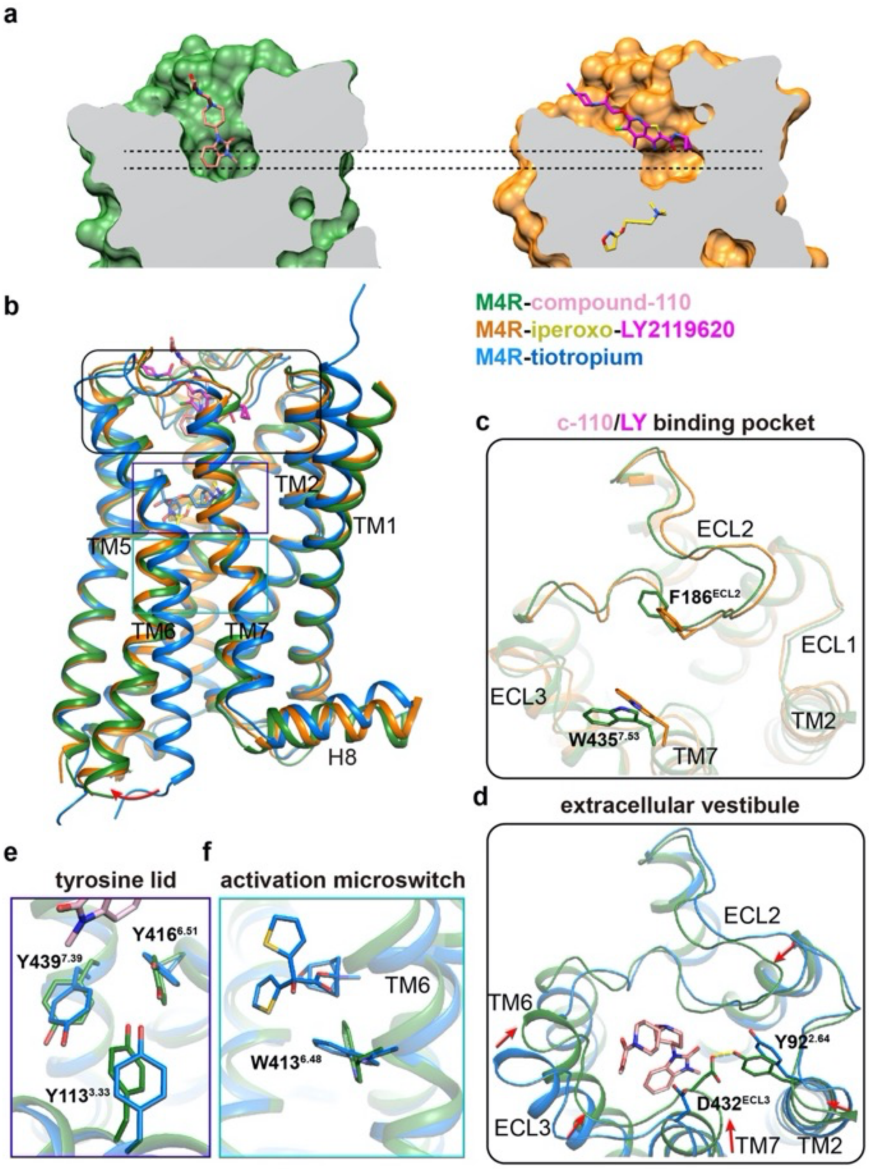

Supplementary Figure 10. Comparison of active M4R-c110 and M4R-ip-LY structures with inactive M4R structure. a) Surface cutaway side views comparing ligand binding positions of compound-110, LY2119620 and iperoxo in M4R structures. **b)** Comparison of inactive M4R (PDB code 5DSG, blue), M4R-c110-G_i (green) and M4R-ip-LY (orange)-G_i structures in sideview. **c)** Conformational change of the extracellular region between the M4R-c110-Gi and M4R-ip-LY structures. **d)** Comparison of the extracellular vestibule in the M4R-c110-G_i and inactive M4R structures. **e)** The relatively closed “tyrosine lid” in the M4R-c110-G_i structure compared to that in the inactive structure. **f)** Rotation of the activation microswitch of Trp413$^{6.48}$ between inactive and active M4R structures.

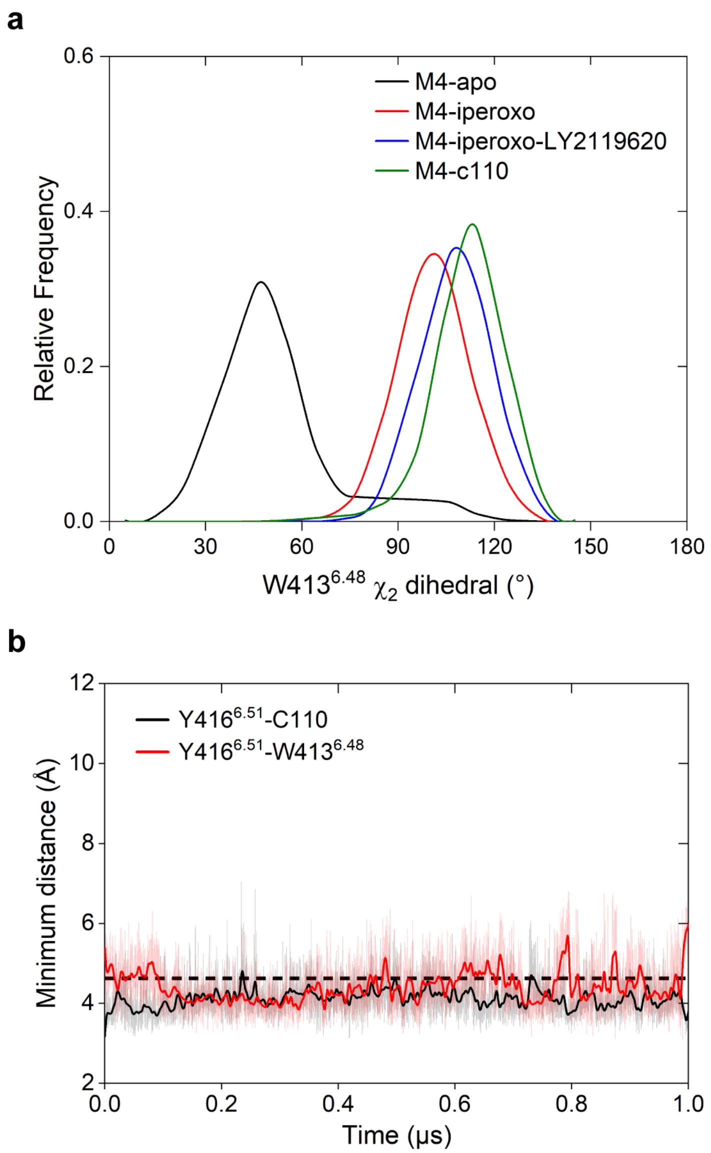

Supplementary Figure 11. a) Distribution of dihedral angle of the Trp413$^{6.48}$ side chain ($\chi2$) during the M4R MD simulations and **b)** distance between Tyr416$^{6.51}$ and Trp413$^{6.48}$ during the M4R-c110 MD simulations.

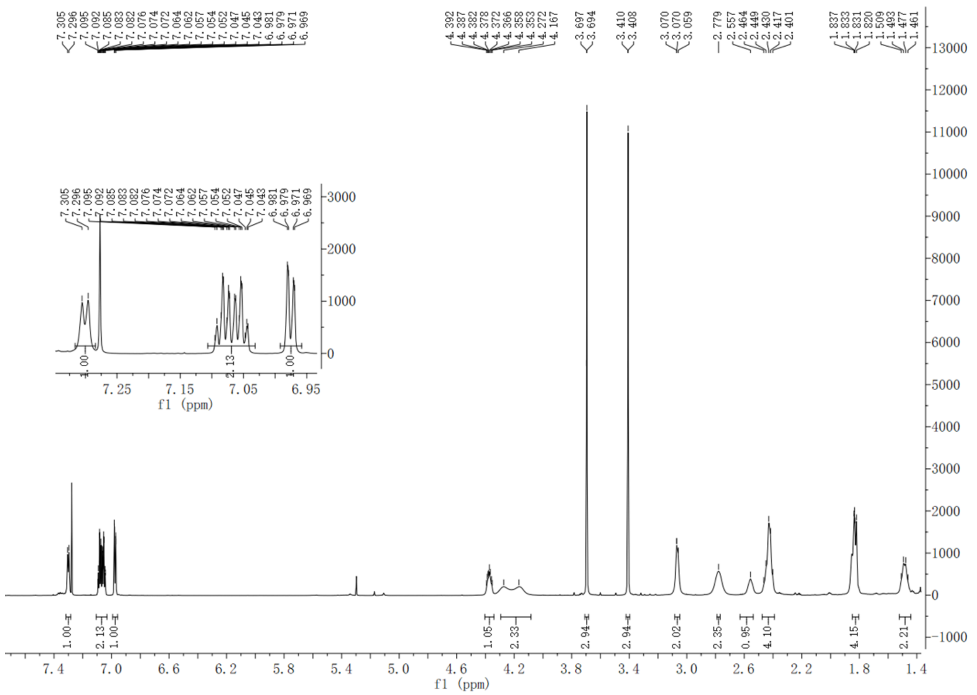

Supplementary Figure 12. ^{1}H-NMR spectra of compound-110

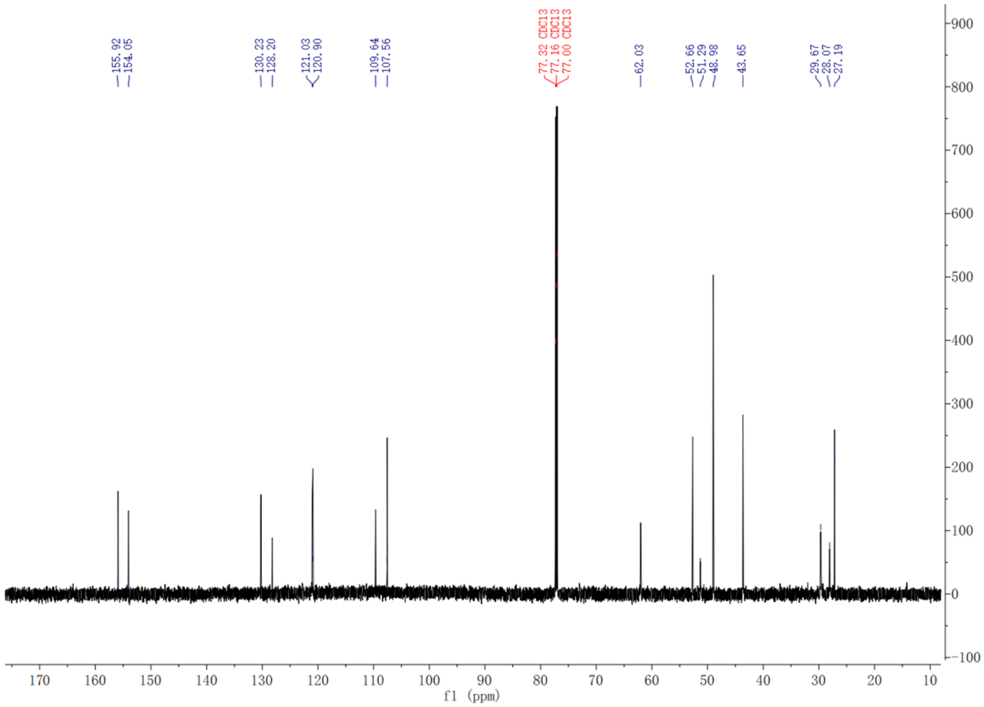

Supplementary Figure 13. ^{13}C-NMR spectra of compound-110

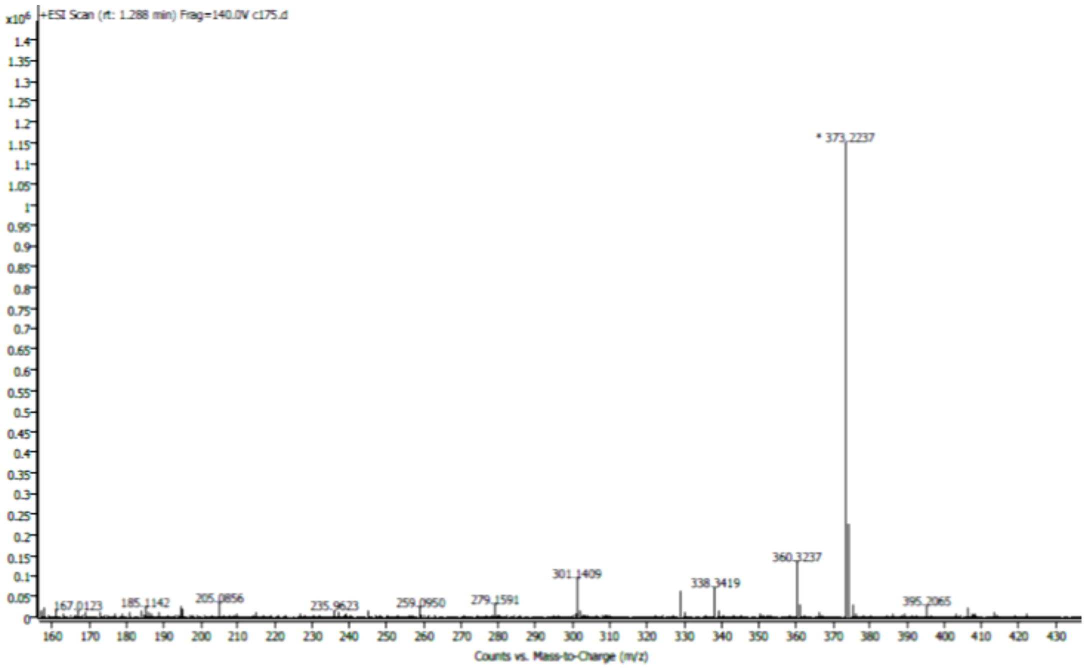

Supplementary Figure 14. HRMS spectra of compound-110

Supplementary Table 1. Cryo-EM data collection, refinement and validation statistics.

	#1 M4R-compound110 -G_i-scFv16	#2 M4R-iperoxo -G_i-scFv16	#3 M4R-ip-LY -G_i-scFv16
**Data collection and processing**			
Magnification	130,000	130,000	130,000
Voltage (kV)	300	300	300
Electron exposure (e-/Å2)	1.333	1.333	1.333
Defocus range (um)	-1.2~-2.0	-1.2~-2.0	-1.2~-2.0
Pixel size (Å)	0.52	0.52	0.52
Symmetry imposed	C1	C1	C1
Final particle images (no.)	78,471	270,892	173,315
Map resolution (Å)	3.6	3.4	3.4
FSC threshold	0.143	0.143	0.143
**Refinement**			
Initial model used (PDB code)	5DSG	5DSG	5DSG
Map sharpening B-factor ($Å^2$)	58.4	64.2	103.1
Model composition			
Non-hydrogen atoms	8584	8539	8574
Protein residues	1122	1122	1122
B-factors ($Å^2$)			
Protein	89.16	114.62	105.93
Ligand/Ion	128.42	-	123.55
RMSDs			
Bond lengths (Å)	0.003	0.003	0.006
Bond angles (°)	0.690	0.660	0.790
Validation			
MolProbity score	1.96	1.79	1.93
Clash score	14.04	13.05	16.35
Rotamer outliers (%)	0.11	0.00	0.00
Ramachandran Plot Statistics (%)			
Favored regions	95.56	97.10	96.83

Allowed regions	4.44	2.90	3.17
Disallowed regions	0.00	0	0

Supplementary Table 2. Effects of the mutations of key residues on compound-110, Ach, iperoxo and LY2119620. E_{max} and pEC_{50} were calculated from concentration-response curves of BRET assay. Data were normalized to the reference ligand iperoxo. ND, not detected. All data are the mean ± SEM (n = 3 independent experiments).

	ACh			LY2119620		
	pEC_{50}	ΔpEC_{50}	E_{max}	pEC_{50}	ΔpEC_{50}	E_{max}
WT	6.99 ± 0.06	0	98 ± 2	6.21 ± 0.10	0	57 ± 1
D112E	6.20 ± 0.01	-0.79	87 ± 4	ND	ND	ND
F186A	6.80 ± 0.08	-0.19	95 ± 3	5.02 ± 0.02	-1.19	76 ± 1
R394A	6.68 ± 0.07	-0.31	94 ± 3	6.15 ± 0.12	-0.06	51 ± 4
Y416A	5.50 ± 0.17	-1.49	75 ± 7	ND	ND	ND
D432A	7.14 ± 0.16	0.15	95 ± 1	6.49 ± 0.19	0.28	63 ± 3
W435A	5.91 ± 0.06	-1.08	88 ± 1	5.10 ± 0.11	-1.11	38 ± 1
Y439A	4.02 ± 0.43	-2.97	74 ± 6	6.30 ± 0.38	0.09	3 ± 2

	Iperoxo		Compound-110		
	pEC_{50}	ΔpEC_{50}	pEC_{50}	ΔpEC_{50}	E_{max}
WT	9.89 ± 0.12	0	6.87 ± 0.07	0	98 ± 3
D112E	9.07 ± 0.03	-0.82	5.84 ± 0.09	-0.97	69 ± 7
F186A	9.68 ± 0.11	-0.21	7.53 ± 0.07	0.66	95 ± 2
R394A	9.54 ± 0.06	-0.35	6.37 ± 0.03	-0.5	74 ± 3
Y416A	7.94 ± 0.28	-1.95	6.59 ± 0.13	-0.28	74 ± 4
D432A	9.93 ± 0.09	0.04	6.52 ± 0.06	-0.35	87 ± 3
W43A	8.88 ± 0.14	-1.01	6.81 ± 0.11	-0.06	69 ± 2
Y439A	6.74 ± 0.10	-3.15	6.86 ± 0.12	-0.01	38 ± 3
