# Supplementary material for: The unconventional activation of the muscarinic acetylcholine receptor M4R by diverse ligands
Source: Nat Commun. 2022 May 23;13:2855. doi: 10.1038/s41467-022-30595-y (PMC9126879; doi:10.1038/s41467-022-30595-y)
Supplement: Supplementary file 3 — Reporting Summary [file 41467_2022_30595_MOESM3_ESM.pdf]

nature research

Corresponding author(s): Tian Hua

Last updated by author(s): May 3, 2022

Reporting Summary

Nature Research wishes to improve the reproducibility of the work that we publish. This form provides structure for consistency and transparency in reporting. For further information on Nature Research policies, see our Editorial Policies and the Editorial Policy Checklist.

Statistics

For all statistical analyses, confirm that the following items are present in the figure legend, table legend, main text, or Methods section.

n/a	Confirmed	
☐	☒	The exact sample size (*n*) for each experimental group/condition, given as a discrete number and unit of measurement
☐	☒	A statement on whether measurements were taken from distinct samples or whether the same sample was measured repeatedly
☒	☐	The statistical test(s) used AND whether they are one- or two-sided<br>*Only common tests should be described solely by name; describe more complex techniques in the Methods section.*
☒	☐	A description of all covariates tested
☒	☐	A description of any assumptions or corrections, such as tests of normality and adjustment for multiple comparisons
☐	☒	A full description of the statistical parameters including central tendency (e.g. means) or other basic estimates (e.g. regression coefficient) AND variation (e.g. standard deviation) or associated estimates of uncertainty (e.g. confidence intervals)
☐	☒	For null hypothesis testing, the test statistic (e.g. *F*, *t*, *r*) with confidence intervals, effect sizes, degrees of freedom and *P* value noted<br>*Give P values as exact values whenever suitable.*
☒	☐	For Bayesian analysis, information on the choice of priors and Markov chain Monte Carlo settings
☒	☐	For hierarchical and complex designs, identification of the appropriate level for tests and full reporting of outcomes
☒	☐	Estimates of effect sizes (e.g. Cohen's *d*, Pearson's *r*), indicating how they were calculated

Our web collection on statistics for biologists contains articles on many of the points above.

Software and code

Policy information about availability of computer code

Data collection	Cryo-EM single particle data was automatically collected on the Titan Krios using serialEM 3.7.3.
Data analysis	cryoSPARC v.2.11, Phenix 1.19.2, COOT 0.8.9.2, PyMOL2.5.0, GraphPad Prism 8.1, Schrödinger Suite 2019-4, 'LigPrep' (Schrödinger), Amber18, Glide 6.9, UCSF Chimera X 0.9, MolProbity.

For manuscripts utilizing custom algorithms or software that are central to the research but not yet described in published literature, software must be made available to editors and reviewers. We strongly encourage code deposition in a community repository (e.g. GitHub). See the Nature Research guidelines for submitting code & software for further information.

Data

Policy information about availability of data

All manuscripts must include a data availability statement. This statement should provide the following information, where applicable:

- Accession codes, unique identifiers, or web links for publicly available datasets
- A list of figures that have associated raw data
- A description of any restrictions on data availability

Data supporting the findings of this manuscript are available from the corresponding authors upon reasonable request. Source data are provided with this paper. The coordinates for M4R-c110-Gi-scFv16, M4R-ip-Gi-scFv16 and M4R-ip-LY-Gi-scFv16 have been deposited in the Protein Data Bank with the accession codes 7V6A, 7V69 and 7V68. The EM maps for M4R-c110-Gi-scFv16, M4R-ip-Gi-scFv16 and M4R-ip-LY-Gi-scFv16 have been deposited in EMDB with the codes EMD-31740, EMD-31739 and EMD-31738, respectively.

Field-specific reporting

Please select the one below that is the best fit for your research. If you are not sure, read the appropriate sections before making your selection.

- [x] Life sciences
- [ ] Behavioural & social sciences
- [ ] Ecological, evolutionary & environmental sciences

For a reference copy of the document with all sections, see nature.com/documents/nr-reporting-summary-flat.pdf

Life sciences study design

All studies must disclose on these points even when the disclosure is negative.

Sample size	No sample-size was calculated. For the functional cell based assays, all assays were performed in 3 or more separate experiments wherein all points were run in duplicate.
Data exclusions	No data were excluded.
Replication	Cell-based signaling assays were independently replicated by two investigators once a week.
Randomization	Drug treatments were performed in dose-response studies on the same set of cells that also received control treatments and vehicle treatments on the same plates. All normalization to control and baseline/vehicle occurred within plate, then averaged among replicates. Drug treatment was randomized on the plate to avoid "plate effects." Wild-type controls were always run in parallel with mutant receptors.
Blinding	There was no blinding in the study.

Reporting for specific materials, systems and methods

We require information from authors about some types of materials, experimental systems and methods used in many studies. Here, indicate whether each material, system or method listed is relevant to your study. If you are not sure if a list item applies to your research, read the appropriate section before selecting a response.

Materials & experimental systems

n/a	Involved in the study	
	x	Antibodies
	x	Eukaryotic cell lines
x		Palaeontology and archaeology
	x	Animals and other organisms
x		Human research participants
x		Clinical data
x		Dual use research of concern

Methods

n/a	Involved in the study	
x		ChIP-seq
x		Flow cytometry
x		MRI-based neuroimaging

Antibodies

Antibodies used	HA Epitope Tag Antibody, Alexa Fluor 488 conjugate (16B12), ThermoFisher
Validation	This antibody has been validated for use in flow cytometry and immunohistochemical staining previously (https://www.thermofisher.com/antibody/product/HA-Tag-Antibody-clone-16B12- Monoclonal/A-21287?pluginName=).

Eukaryotic cell lines

Policy information about cell lines

Cell line source(s)	HTLA cells (an HEK293 cell line stably expressing a tTA-dependent luciferase reporter and a -arrestin2-TEV fusion gene) were generously provided by Richard Axel's lab. HEK 293T cells were provided by ATCC (CRL-11268).
Authentication	The cells expressing wild-type or mutant M4R were authenticated by confocal microscopy and flow cytometry using the antibodies described above.
Mycoplasma contamination	All cells have been tested as negative for mycoplasma contamination.
Commonly misidentified lines (See ICLAC register)	No commonly misidentified cell lines were used.

Animals and other organisms

Policy information about studies involving animals; ARRIVE guidelines recommended for reporting animal research

Laboratory animals	C57B6/J male mice purchased from Shanghai SLAC Co.,Ltd were raised to 10~12 weeks old for animal tests.
Wild animals	No
Field-collected samples	No
Ethics oversight	All animal experiments were performed in accordance with the protocol (SIBCB-S375-1912-027) approved by the Animal Care and Use Committee of the Center for Excellence in Molecular Cell Science, Chinese Academy of Sciences.

Note that full information on the approval of the study protocol must also be provided in the manuscript.
